# 2625. Characteristics and Outcomes of COVID-19 in Patients with a Left Ventricular Assist Device

**DOI:** 10.1093/ofid/ofad500.2238

**Published:** 2023-11-27

**Authors:** Jorge Fleites, Yoichiro Natori, Jacques Simkins, Hoda Butrous, Jospeh Bauerlein, Anita Phancao, Matthias Loebe, Mrudula Munagala, Shweta Anjan

**Affiliations:** University of Miami Miller School of Medicine, Miami, Florida; Miami Transplant Institute, Miami, Florida; Miami Transplant Institute, Miami, Florida; Miami Transplant Institute, Miami, Florida; Miami Transplant Institute, Miami, Florida; Miami Transplant Institute, Miami, Florida; Miami Transplant Institute, Miami, Florida; Miami Transplant Institute, Miami, Florida; University of Miami Miller School of Medicine and Miami Transplant Institute, Jackson Health System, Miami, Florida

## Abstract

**Background:**

Patients with left ventricular assist devices (LVAD) infected with SARS-CoV-2 may be at increased risk of complications and mortality. The purpose of the study was to analyze the characteristics and outcomes of LVAD patients with SARS-CoV-2 infection at our center.

**Methods:**

A retrospective review of LVAD patients diagnosed with COVID-19 via PCR between March 1st, 2020, and March 1st, 2023, was conducted. Patient demographics, clinical, laboratory, and imaging data were collected. We performed descriptive statistical analysis on the data obtained.

**Results:**

During the study period, we were actively following 130 LVAD patients, 34 (26.2%) developed COVID-19. Our cohort consisted of 27 males (79.4%) with a median age of 63.5 years and median BMI of 27.6 (Table 1). Eighteen and 12 self-identified as African American (52.9%) and Hispanic (35.3%), respectively. The common comorbidities included hypertension (94.1%), hyperlipidemia (79.4%), atrial fibrillation (67.4%), and chronic kidney disease (61.8%). The most common presenting symptoms were shortness of breath (41.2%), cough (35.3%), and fever (29.4%). Nine (26.5%) patients were asymptomatic. Twenty-eight (82.4%) and 16 (57.1%) were admitted to the hospital and ICU, respectively (Table 2). Ten (35.7%) and 2 (7.1%) patients required supplemental oxygen via nasal canula, and mechanical ventilation, respectively. Most of the hospitalized patients were managed with Remdesivir (71.4%) and systemic corticosteroids (42.9%). No complications of pump hemolysis or thrombosis, or systemic embolisms were noted. Two deaths were reported: one patient (2.9%) died while in the hospital from COVID-19, and another patient died 16 months later secondary to acute respiratory failure in the setting of post-COVID pulmonary fibrosis.

**Figure:**
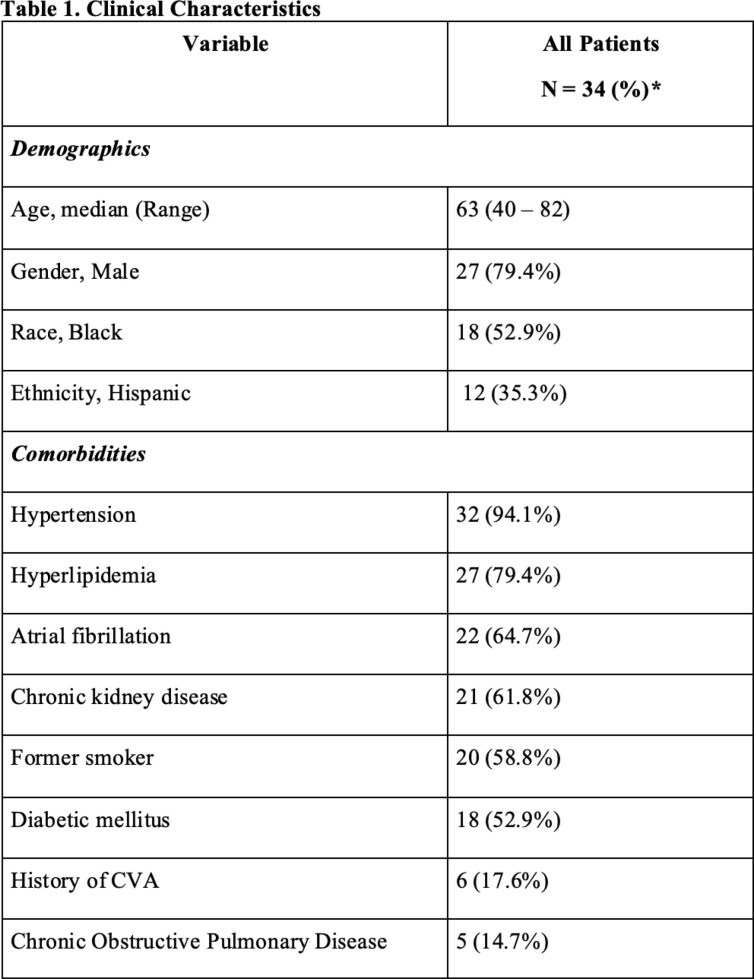

**Figure:**
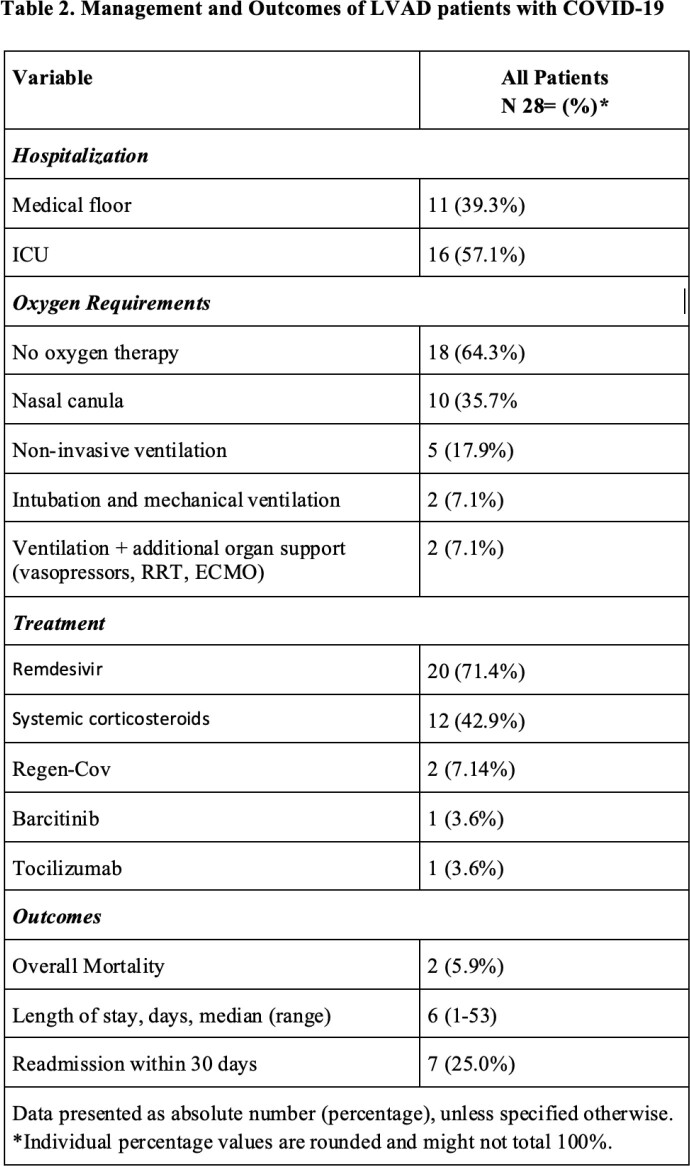

**Conclusion:**

This is the largest single center study analyzing outcomes of COVID-19 in LVAD patients to date. Our cohort experienced a lower mortality rate from COVID-19 infection compared to prior studies. Larger studies are needed to guide management strategies and determine optimal timing for heart transplant after COVID-19.

**Disclosures:**

**All Authors**: No reported disclosures

